# Transient Hiccups Associated with Oral Dexamethasone

**DOI:** 10.1155/2013/426178

**Published:** 2013-10-09

**Authors:** Mark E. Peacock

**Affiliations:** Department of Periodontics, Georgia Regents University College of Dental Medicine, 1120 15th Street, Augusta, GA 30912-1241, USA

## Abstract

Hiccups, or singulata (hiccup is singultus), are commonly experienced by most people at one time or another and are usually brief and self-limiting. Although pharmacotherapeutic agents are not generally considered causal in the etiology of hiccups, many clinicians empirically associate episodic hiccups in their patients as being drug induced. The two classes of drugs most often cited as causing hiccups are corticosteroids and benzodiazepines. This report involved a patient who was given preoperative dexamethasone and developed hiccups before anesthesia and surgery commenced. He at no time was in distress, and the surgical procedure was completed without complication. By the second postsurgical day his hiccups were resolved completely. Although the association may be anecdotal, many clinicians consider hiccups a potential side effect of steroid therapy, especially high doses of steroids. Of interest in this case is the relatively low dose of corticosteroid used, albeit apparently linked to his hiccups. Practitioners should be aware of this potential condition.

## 1. Introduction

Hiccups, or singulata (hiccup is singultus), are very common and are experienced by most people at one time or another. They are usually brief and self-limiting but may become prolonged in some patients [1]. Hiccups that linger on for some time may become worrisome to the postoperative patient, thus hindering their nutritional and sleep needs [2, 3]. 

Hiccups are sudden, uncontrolled contractions of the diaphragm, followed by immediate inspiration and closure of the glottis over the trachea, producing the “hiccup” sound [4]. The classification of hiccups is as follows: up to 48 hours, acute or transient; longer than 48 hours, persistent; and more than a month or two, intractable [5]. The frequency of hiccups in males and females is equivalent, although intractable hiccups occur at a much higher rate in men [6, 7]. The exact etiology of the hiccup is unknown, but the neural process involves the reflex arc consisting of the afferent limb, the center, and the efferent limb [8, 9]. The afferent limb contains the phrenic and vagus nerves together with the sympathetic chain from T6 to T12. The center is linked to the afferent and efferent limbs and occupies a nonspecific location somewhere between C3 and C5. The efferent limb includes the phrenic nerve, accessory respiratory muscles, the glottis, and autonomic processes involving the medullary reticular formation and hypothalamus [4, 10]. One review proposed that the hiccup reflex arc is a myoclonic action and not a true reflex [11]. 

Medical conditions that have been associated with the development of hiccups include gastrointestinal, neurological, pulmonary, psychogenic, cardiovascular, metabolic, anesthesia related, and drug induced conditions [3, 4, 8, 12, 13]. Using a strict standard, drugs have not been proven to be a common cause of hiccups [7, 14]. Nevertheless, many clinicians have alluded to various medications as triggering the hiccup reflex [1, 3, 6, 13, 15–24]. The following case describes a patient who experienced transient hiccups following oral presurgical administration of dexamethasone.

## 2. Case Report

A 40-year-old male with an unremarkable medical history presented for surgery to place an implant. He was in excellent health, did not take any medications, and was not allergic to any drugs. The patient had taken a single prophylactic dose of 8 mg oral dexamethasone approximately 1 hour earlier. After presurgical vital signs were taken, and before any other medication (sedation, local anesthesia) was administered, the patient developed intermittent bouts of hiccups at a rate of roughly 5 to 7 per minute. He was in no distress and wanted to continue the procedure. Oral triazolam 0.50 mg was given, and by the time the surgery started, the episodic hiccups were reduced greatly allowing the implant to be placed uneventfully. By the time the patient was ready to be escorted from the clinic, the hiccups had returned at about the same rate they occurred preoperatively. He was given postoperative instructions and reassurances and followed up telephonically the next day, where he reported that by late afternoon (32 hours) the rate of hiccup episodes was reduced. The patient's hiccups resolved completely by 42 hours after he took the dexamethasone. At the 1-week postoperative appointment, the incident was reviewed with the patient and counseling was given on the suspected drug-induced cause of the transient hiccups for his future reference/benefit.

## 3. Discussion

There are few reports in the literature on dexamethasone-induced hiccups and none in the dental literature [6, 15, 18, 23, 24]. Other cases of corticosteroid-induced hiccups have been reported [1, 25], and Dickerman et al. have described the first cases of anabolic steroid-induced hiccups [16, 17]. The only other adverse reaction to steroids found in the dental literature was a case of episodic psychiatric disturbance (cognitive dysfunction) in an 18-year-old female who had taken dexamethasone briefly [26]. The author would be remiss not to mention another suspected dexamethasone-induced transient hiccups case he came across years earlier, but, because other drugs were also given intravenously at the same time, it could not be confirmed.

Corticosteroids and benzodiazepines are the drug groups referenced most frequently in the literature as being associated with hiccups (see the following list), although Thompson and Landry state that there is not sufficient proof that any drug can be considered as definitely causing hiccups [14]. Souadjian and Cain reviewed 220 cases of protracted hiccups and did not mention any medication in the etiology of hiccups [7]. Garvey, who looked at postoperative cases of hiccups, came to the logical conclusion that the etiologic factor was probably drug related [3]; however, she also recounted that the intubation itself may be a contributing factor [27].

*Drugs Possibly Associated with Triggering Hiccups*:

 Steroids (dexamethasone, methylprednisolone, oxandrolone, and progesterone)
 Benzodiazepines (midazolam, lormetazepam, and lorazepam)
 Barbiturates (methohexital)
 Antibiotics (azithromycin)
 Phenothiazines (perphenazine)
 Opioids (hydrocodone)
 Alcohol.

The case described here was mild and short term and, even though somewhat inconvenient to the patient, was in practice, clinically insignificant. Hiccups that become persistent or intractable however can interfere with a patient's daily activities and cause them to seek medical assistance. There are various reports in the literature of different treatments for protracted hiccups, including pharmacologic agents [4, 5, 8, 18, 22, 28–34]. Chlorpromazine is at present the only medication approved by the FDA for the treatment of hiccups, although many practitioners have reported less than desirable results with this drug [6, 17, 29]. 

Baclofen has been shown to successfully treat chronic hiccups [3, 4, 19, 30, 34], and promising results have been attained with the use of gabapentin alone [31] or as an add-on to combination therapy [5, 32]. 

The evidence for medication-induced hiccups may be empirical, yet for many the association is strong enough that clinicians should take notice. This is especially true for treatments involving steroids [35], drugs that are commonly used in medicine, including dental medicine. Being able to recognize the potential “drug-hiccup link” will better prepare health care practitioners manage any unexpected complications.

## 4. Conclusions

There are many uses for steroids in medicine and dentistry, and clinicians should be attentive to any possible side effects of medications prescribed. This paper and case explain the correlation between hiccups and steroid treatment in the perioperative setting. Although drug-induced hiccups have not been absolutely confirmed with controlled studies, the incidence is sufficient enough to raise questions by many practitioners. Fortunately, most cases of corticosteroid-related hiccups appear to be transient and usually end after the drug is withdrawn.
